# Add-on exercise interventions for smoking cessation in people with mental illness: a systematic review and meta-analysis

**DOI:** 10.1186/s13102-022-00498-y

**Published:** 2022-06-21

**Authors:** Stefanie E. Schöttl, Martin Niedermeier, Prisca Kopp-Wilfling, Anika Frühauf, Carina S. Bichler, Monika Edlinger, Bernhard Holzner, Martin Kopp

**Affiliations:** 1 Department of Sport Science, University of Innsbruck, Fürstenweg 185, 6020 Innsbruck, Austria; 2 Department of Psychiatry, Psychotherapy and Psychosomatics, Psychiatry I, Medical University of Innsbruck, Anichstraße 35, 6020 Innsbruck, Austria

**Keywords:** Systematic review, Meta-analysis, Smoking, Smoking cessation, Exercise, Physical activity, Mental illness, Mental disorder

## Abstract

**Background:**

Smoking is the most common substance use disorder among people with mental illness. In contrast to people without mental illness, among whom the proportion of smokers has declined in recent decades, the proportion of smokers among people with mental illness remains high. There is a growing body of literature suggesting the use of exercise interventions in combination with smoking cessation in people without mental illness, but to our knowledge the available studies on this treatment option in people with mental illness have not been systematically reviewed. Therefore, this systematic review and meta-analysis aims to assess the effectiveness of exercise interventions as an adjunctive treatment for smoking cessation in people with mental illness.

**Methods:**

Electronic databases (PubMed, Web of Science, PsycInfo, Sport Discus and Base) were searched for randomised controlled trials and prospective single-group studies that investigated exercise interventions in combination with smoking cessation programmes alone or in comparison with a control group in people with mental illness. A meta-analysis using the Mantel–Haenszel fixed-effect model was conducted to estimate the overall effect of treatment on smoking cessation (abstinence rate at the end of the intervention and at 6-month follow-up).

**Results:**

Six studies, five randomised controlled trials and one study with a prospective single-group design, were included in the systematic review and four randomised controlled trials were included in the meta-analysis. The meta-analysis found a significantly higher abstinence rate after additional exercise at the end of the intervention [risk ratio (RR) 1.48, 95% confidence interval (CI) 1.13–1.94], but not at the 6-month follow-up (RR 1.34, 95% CI 0.89–2.04).

**Conclusions:**

Exercise appears to be an effective adjunctive therapy to temporarily increase abstinence rates in individuals with mental illness at the end of the intervention. However, due to the small number of included studies and some risk of bias in the included studies, the results should be treated with caution. Therefore, future studies with larger samples are needed to provide a more accurate estimate of the effect in people with mental illness.

*Registration* The systematic review and meta-analysis were registered in the International Prospective Register of Systematic Reviews (PROSPERO) (registration number: CRD42020178630).

## Background

People with mental illness (MI) such as schizophrenia, depression, bipolar disorder, or anxiety disorders show a lower quality of life, poorer physical health and higher mortality than individuals without MI [1–4]. Cardiovascular disease is the main cause of death in people with MI, often caused by health risk behaviours and cardiovascular risk factors such as obesity, hypertension, diabetes mellitus, poor diet, insufficient physical activity (PA), and smoking [3–5]. In particular, smoking, the most common substance use disorder in people with MI, is an important modifiable risk factor and contributes to the high mortality rate [6, 7]. Depending on the severity and type of mental disorders and diagnosis, the prevalence of smoking in people with MI is two to four times higher than in the rest of the population [7–9]. It has also been shown that heavy nicotine dependence and heavy smoking are more common in smokers with schizophrenia than among smokers in the general population [10].

While the proportion of smokers in the general population has decreased in recent decades [11], the proportion of smokers with MI remains high [6, 12]. Two main factors are discussed in the literature as reasons for this discrepancy: first, smoking cessation efforts are mainly focused on the general population, while individuals with MI are a difficult group to reach for health interventions [12, 13]. Second, nicotine dependence is more pronounced in people with MI, making quitting particularly difficult [14, 15]. In a study by Leon et al. [16], the number of smokers who successfully quit was significantly lower in patients with mood disorders and schizophrenia than in the control group without mental disorders. At the current state of research, people with MI are concerned about the health effects of smoking and their motivation to quit smoking is similar to those of the general population [17, 18]. Nevertheless, the readiness of individuals with MI to participate in smoking cessation programmes is lower, as most patients reported that they have reduced confidence in their ability to quit [18]. Therefore, it is important to develop additional and effective smoking cessation strategies to help people with MI quit [6].

For both groups of smokers, people with and without MI, common interventions such as pharmacological interventions, behavioural therapy, and psychosocial support are used for smoking cessation [6, 7, 19, 20]. There is a growing body of literature examining the use of exercise interventions as add-on therapy to smoking cessation programmes in people without MI [21–25], but less evidence is available for this treatment option in individuals with MI [7, 19]. Exercise as an additional treatment is not only a cost-effective and easily accessible treatment, but may also have many general health benefits for people with and without MI [26, 27]. Acute bouts of exercise can help to mitigate withdrawal symptoms and craving for cigarettes [28, 29], and help people to pass the time when they are bored [18]. Regular exercise can also improve cognitive performance [30, 31], positively change mood, and reduce negative feelings and depressive symptoms [32]. Numerous studies indicate positive long-term effects of exercise on the cardiovascular system [33], well-being [34, 35], and quality of life [36, 37].

Some reviews and meta-analyses have already focused on acute effects of physical activity on cigarette craving [28, 38, 39], and on effectiveness of exercise interventions alone or in combination with smoking cessation programmes in people without MI [26, 27]. However, these findings in people without MI cannot be generalised to people with MI, because individuals with MI differ from individuals without MI in the following aspects, which should be considered in an intervention. People with MI (a) are already under treatment, with a focus on treating the mental disorder. Some healthcare professionals have concerns, that an additional smoking cessation treatment may interfere with the treatment of the mental disorder [15]. Furthermore, individuals with MI (b) often take psychiatric medications that can cause undesirable side effects [15, 40]. This may result in limited willingness of people with MI to take additional smoking cessation medications e.g. bupropion or varenicline. People with MI (c) may also have comorbid substance abuse, (d) may interpret smoking as a form of self-medication, (e) may be in an unstable phase of mental health, where quitting or reducing smoking may be particularly more challenging than in a stable phase of mental health, (f) and are often facing lower socioeconomic status, low social support, limited education, poverty, and unemployment [15, 40, 41]. While these disadvantages are partly traced back to common misconceptions among healthcare professionals, this also leads to a particular need for smoking cessation treatment in patients with MI [15].

Although there are some preliminary findings that exercise interventions in combination with smoking cessation counselling can have a positive impact on smoking abstinence rates in people with MI [42], to our knowledge, the available studies on exercise interventions as an add-on therapy to smoking cessation in people with MI have not been systematically reviewed. The authors' aim was therefore to fill this gap by conducting a systematic review of the scientific literature.

The aim of this systematic review and meta-analysis is to evaluate the effectiveness of exercise interventions as an add-on therapy to smoking cessation in people with MI. The following research questions are addressed: (1) Do exercise interventions added to smoking cessation treatments increase smoking abstinence rates among individuals with mental illness? (2) Do exercise interventions as add-on therapy to smoking cessation treatments have a positive impact on craving, withdrawal symptoms, psychological symptoms, quality of life, and physical activity for people with mental illness? (3) How do intervention studies differ by study characteristics (e.g. sample, intervention and control condition, outcomes)?

## Methods

The PRISMA (Preferred Reporting Items for Systematic Reviews and Meta-Analyses) guidelines were applied for conducting and reporting this systematic review and meta-analysis [43]. The systematic review and meta-analysis were registered in the International Prospective Register of Systematic Reviews (PROSPERO, www.crd.york.ac.uk/PROSPERO/) on July 14th 2020 (registration number: CRD42020178630).

### Eligibility criteria

#### Types of studies and intervention

Randomised controlled trials (RCTs) examined exercise interventions as add-on therapy to smoking cessation compared to smoking cessation treatment alone or smoking cessation treatment combined to a control condition (without exercise) among people with MI were included in this review. Studies with a prospective single-group design dealing with exercise interventions in combination with smoking cessation but without a control group were also included in the systematic review but not in the meta-analysis.

The entire programme had to be conducted regularly for at least 5 weeks that an effect of the programme on smoking abstinence rate could be observed. The individual components of the programmes (smoking cessation treatment, exercise intervention, control condition) also had to be implemented regularly with multiple sessions, either several times per week for a shorter period (for at least 3 weeks) or once per week for a longer period (for at least 5 weeks). A further requirement for the included studies were assessment points at the baseline and after the treatment. Studies with follow-up assessments or measurements during the interventions were also included in the review.

There were no restrictions on the type and intensity of exercise intervention, the type of smoking cessation programme, and the type of control group. Different approaches for smoking cessation treatment may have been used in the included studies, such as behavioural therapy, psychosocial support, pharmacotherapy or nicotine replacement therapy. Exercise interventions could have consisted of any type of sports e.g. walking, running, cycling, fitness at low to high intensity. Only studies in which participants were physically active as a part of the exercise intervention could be included in the review. On the one hand, the exercise programme could be delivered in a setting within the study, e.g. supervised exercise by an instructor. On the other hand, participants of the included studies could also choose the location of the exercise. The requirement for this was that the studies verified the execution of the exercise programme e.g. by surveys, diary entries or objective measurement instruments. Studies whose participants were not required to actively participate in the exercise interventions and only were informed about the topic “exercise”, could not be included in the review. If the exercise intervention did not consist of multiple sessions, as mentioned previously, the study was not considered suitable for this review. The programme of the control group could include either a smoking cessation programme alone or a smoking cessation programme in combination with other control conditions, e.g., relaxation, wellness programme, or equivalent social contact. If PA was also a part of the control group in a study, that study was excluded from the meta-analysis but included in the systematic-review.

Studies that included exercise and smoking in a multicomponent programme, e.g. health interventions, well-being programmes, change trials, healthy lifestyle interventions, or cardiovascular risk reduction programmes, were excluded. As exercise interventions and smoking cessation programmes are part of many other interventions, e.g., alcohol reduction, healthy eating, diet, weight management, sleep quality, smoking reduction or abstinence cannot be explained by the effect of exercise.

#### Types of participants

Studies that examined the adult population (≥ 18 years) were included in the review. An important requirement for the review was that the included studies examined smokers (≥ 5 cigarettes/day) with MI. Participants in the included studies had to have any diagnosed mental disorder at baseline, such as schizophrenia or schizoaffective disorder, bipolar disorder, depression, or anxiety disorders. Trials investigating people with elevated anxiety sensitivity were also included in the review, as elevated anxiety sensitivity may be related to emotional disorders and thus may be an indication of the onset of a MI [46]. According to Zvolensky et al. [46], “anxiety sensitivity reflects a relatively stable individual difference factor that predisposes individuals to the development of anxiety/depressive problems by amplifying negative mood states”. Smoking is also more prevalent and more difficult to reduce or quit in people with anxiety disorders and anxiety sensitivity than in the general population [44–46]. In the included studies, the diagnosis of MI was made by using a diagnostic interview or questionnaires, or by consulting psychiatrists who were responsible for the patients. Studies that did not target people with MI but recorded depression and anxiety using questionnaires during the programme were excluded.

#### Types of outcome measures

The primary outcome of the present review was abstinence from smoking which could be indicated by several parameters in the included studies: (a) self-reported nicotine abstinence rate e.g. 7-day point prevalence abstinence, prolonged abstinence, continuous abstinence, (b) self-reported number of cigarettes, or (c) biochemical carbon monoxide concentration test, saliva or urine cotinine. If abstinence from smoking was not reported in a study, nicotine dependence, measured with the FTND (Fagerström Test for Nicotine Dependence), was used to show the change in smoking.

Secondary outcomes were (a) smoking-related outcomes, indicated by self-reported questionnaires on craving, withdrawal symptoms, motivation to quit smoking, or smoking self-efficacy, (b) physical activity assessed by objective instruments (e.g. accelerometer) or by self-reported, validated questionnaires, (c) symptom severity of diagnosed MI measured by validated questionnaires, (d) affective states assessed by validated questionnaires (e.g. PANAS), and (e) treatment adherence documented by frequency of participation in the entire programme (smoking cessation + exercise programme or smoking cessation + control condition).

To examine treatment-related changes in primary and secondary outcomes, the different variables should have been recorded at baseline, post-intervention, follow-up, or between the intervention.

### Information sources and search

Online searches for literature were conducted in the electronic databases PubMed, Web of Science, PsycInfo, Sport Discus, and Base. The following search terms were used: ”exercise” OR” physical activity “ OR” sport “ AND” smoking “ OR” smoking cessation “ AND” mental illness “ OR “mental disorder” OR” depression “ OR” anxiety “ OR” schizophrenia “. A secondary search was conducted in unpublished literature such as master’s-theses and dissertations. In addition, Clinical Trials.gov was searched for ongoing or recently completed studies. In case of a suitable ongoing study, the corresponding author of the trial was contacted. PROSPERO was also searched for ongoing and recently completed systematic reviews and meta-analyses. When relevant studies were identified, the authors checked the reference lists for other relevant cited papers. Personnel files and publications of relevant authors on the topic were hand-searched to avoid overlooking relevant studies. The literature search was continued and updated until the review was completed. The search was limited to the English and German languages and to human subjects. There were no restrictions on the date of publication.

### Study selection, data collection, and extraction

All articles from all databases identified by the search, including title, abstract and full text if available, were uploaded into Distiller Systematic Review Software (Evidence Partners, Ottawa, Canada). Afterwards, all duplicates were removed. Two authors (SS, MN) then reviewed the titles of potentially relevant studies and sorted them out if they were not suitable. In a next step, an abstract screening of the remaining studies was performed, followed by a full text screening. At each stage of screening (title, abstract, full text), two authors (SS, MN) independently undertook the eligibility assessment according to the inclusion and exclusion criteria. After abstract and full text screening, the level of agreement between the two authors was checked. If disagreements between the two authors could not be resolved through discussion, a third independent author (MK) was consulted to make a final decision.

After screening, one author (SS) extracted data from all included studies and a second author (MN) verified and completed data extractions. Discrepancies in data extraction were discussed between the authors. If agreement could not be reached, a third author (MK) assisted in the decision-making process. For each included study, data were extracted according to the following criteria: (a) general information about study (title, author, year, country, design); (b) characteristics of sample (recruitment, age, gender, sample size, mental disorder, smoking status); (c) details of interventions (type, content, duration, frequency, intensity, attendance); (d) outcomes (survey and measuring method, point of assessments, follow-up, details of outcomes).

### Risk of bias in individual studies

The risk of bias and quality of included RCTs were assessed using the revised Cochrane risk of bias tool for randomised trials (RoB 2) [47]. For each integrated study, two authors independently answered the signaling questions within each bias domain and rated each domain as being at “high risk”, “low risk” or with “some concerns”. Discrepancies in the judgement of the five domains were discussed. If all domains were rated as low risk, the study was considered as low risk of bias. If the assessment included one or more domains of some concerns but no high risk, the study was classified as having some concerns. A study was categorized as high risk of bias, if one or more domains were found to be at high risk.

The ROBINS-I tool (Risk of Bias In Non-randomised Studies—of Interventions) was used [48] to evaluate the risk of bias in prospective sing-group studies. This tool can be used to assess the risk of bias in different types of NRSI (non-randomised studies of interventions) such as uncontrolled before-after studies [49]. In the ROBINS-I tool, seven domains, covering all types of bias, were judged. In contrast to the risk-of-bias assessment of the RoB 2 tool, the domain and overall assessment can be rated at “low”, “moderate”, “serious” or “critical” risk of bias, while there is an additional option of “no information” for the domain judgement [49].

### Data synthesis

A systematic narrative synthesis of the literature was conducted. Characteristics, primary outcomes and statistically significant secondary outcomes are summarised and presented in the text and in tables. The narrative overview shows the relationship and results within and between the selected studies.

In addition, a meta-analysis was conducted using Review Manager Version 5.4.1 (The Cochrane Collaboration, 2020). Pooled risk ratios (RR) with 95% confidence intervals (CI) were calculated to estimate the overall treatment effect on smoking abstinence at two time-points (end of intervention and 6-month follow-up). For the RR, the number of abstinent participants in the treatment and control groups at two time-points and the total number of participants randomised to the treatment and control groups were used. If the abstinence rate was given in percentage, it was converted. The Mantel–Haenszel fixed-effect model was used for the analysis because the number of participants was small and there was no evidence for heterogeneity [50]. Statistical heterogeneity was measured using Higgins I^2^ [51]. For the meta-analysis, the data of the intention-to-treat (ITT) analysis of the respective studies were used [52, 53].

Due to the small number of included studies, we did not exclude studies with a high risk of bias from the meta-analysis. For the same reason, no subgroup analyses and no sensitivity analyses were conducted.

## Results

### Results of the search

**Fig. 1:**
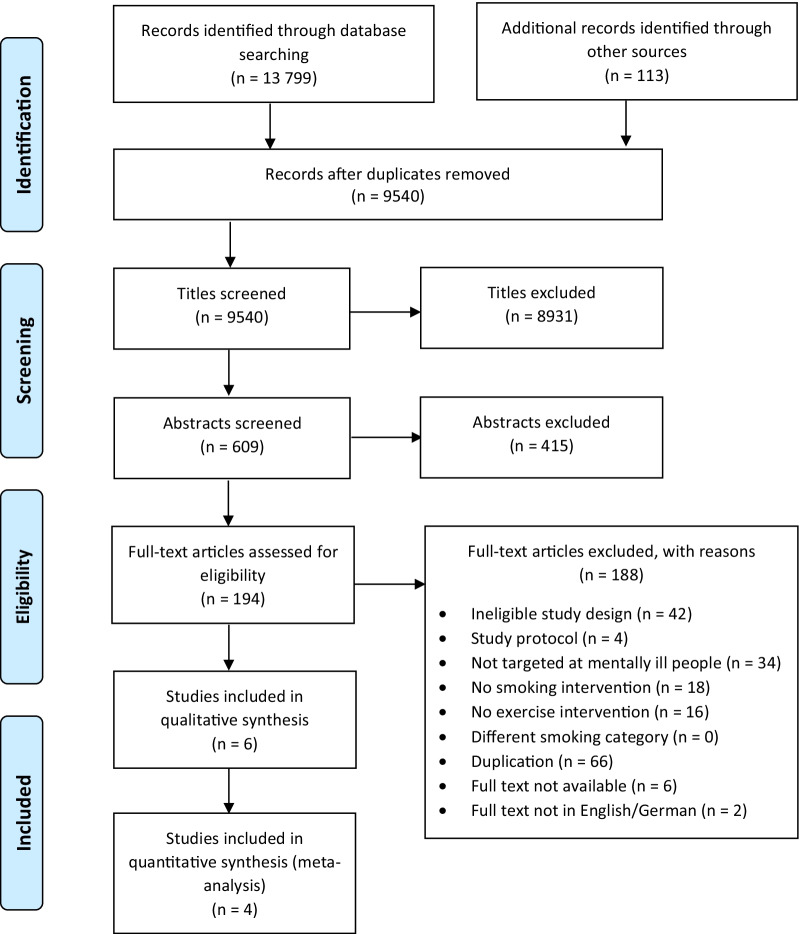
Prisma Flow Diagram of literature search and study selection

A total of 13 911 records were identified through database searches and other sources (identified records from reference lists: 23). After duplicates were removed, 8931 records of the remaining 9540 records were excluded based on the title. Afterwards the abstracts of the remaining 609 articles were screened and 415 records were excluded. As a next step, full-texts of 194 records were assessed for eligibility. 188 articles were excluded according to various reasons (see Fig. 1). Six studies [42, 54–58] met the inclusion criteria for the qualitative synthesis and four studies [42, 55, 56, 58] were included in the meta-analysis. The paper of Zvolensky et al. [59] was excluded from the review during full text screening. The paper presents further secondary outcomes to the original study by Smits et al. [56]. Since further outcomes were not relevant to our review and the study by Smits et al. [56] provides sufficient primary and secondary outcomes, an exclusion of the paper was decided.

### Included studies and study characteristics

Six studies by four different authors were identified during the screening process and included in the systematic review [42, 54–58]. The trials were published in five different journals and within the last 12 years, with the oldest study published in 2009 [58]. Two studies were conducted in France [54, 55] and four in the USA [42, 56–58]. The systematic review includes five RCTs [42, 55–58] and a prospective single-group study [54]. Two of the included studies are described as feasibility studies [54, 58] and two as pilot trials [42, 55]. The detailed study characteristics are summarized in Table 1.

**Table 1:** Study characteristics

Study/design	Sample	Intervention	Control condition	Measuring points	Survey methods
Bernard et al. [54] Single group prospective design	n = 12 Adults (both sexes) with schizophrenia or schizoaffective disorder Age: 45.7 years	8-week group program: a) “smoking reduction” group: counseling intervention based on behaviour change (5 × 75 min) b) exercise sessions: walking (moderate intensity) (3 × 90 min)	–	Baseline, end of intervention, 6-week follow-up	Number of cigarettes, CO concentration, FTND, SEQ-12, Q-MAT, HADS
Bernard et al. [55] RCT	n = 70 (EX: 35, CO:35) Adults (both sexes) with depression Age: 48.5 years	8-week group program: a1) initial individual session: brief counseling and 12-week prescription for NRT (patches, gums) or varenicline a2) standard smoking cessation treatment: 10 counseling sessions with different BCTs (40 min) (weeks 1–2: biweekly, weeks 3–8: weekly) b) group exercise program: two supervised sessions (40-min ergometer, 60–85% maximum heart rate) in first two weeks; one supervised session and one home-based session (40-min walking, cycling or running) in weeks 3–8	c) health education + a1)	Baseline, end of intervention, 12-, 24-, 52-week follow-ups	Number of cigarettes, continuous abstinence rate, CO concentration, FTND, HADS, SEQ-12, TCQ-12, PA (accelerometer, IPAQ-SF), SF-12, PSPP, 6MWT
Patten et al. [42] RCT	n = 30 (EX:15, CO:15) Female adults with depression Age: 37.5 years	12-week individual-based program: a) behavioural smoking cessation counseling (one session each week, 15–20 min) and pharmacotherapy with nicotine patches b) exercise: aerobic activity (cardiovascular equipment of choice) (gradually progression from moderate to vigorous intensity) (three 30–40 min sessions per week)	c) health education + a)	Baseline, end of intervention, 6-month post-target quit date	Number of cigarettes, seven-day point prevalence abstinence rate, saliva cotinine, FTND, PA (maximal oxygen consumption, accelerometer), PHQ-9
Smits et al. [56] RCT	n = 136 (EX: 72, CO: 64) Adults (both sexes) with anxiety sensitivity Age: 44.2 years	15-week group program: a) standard treatment for smoking cessation: 7 weekly 60-min sessions of CBT and subsequently optional NRT patches b) exercise: treadmill (progress exercise intensity to a vigorous level) (three weekly 35-min sessions)	c) wellness education + a)	Baseline, end of intervention, 4- and 6-months post-target quit date	Number of cigarettes, seven-day point prevalence and prolonged abstinence rate, CO concentration, saliva cotinine, FTND, ASI-16, ASI-3, IDAS
Smits et al. [57] RCT	n = 150 (EX1: 77, CO:73) Adults (both sexes) with high anxiety sensitivity Age: 38.6 years	15-week individual program: a) behavioral smoking cessation counseling: Texas Tobacco Quitline (week 3–7) and NRT (week 6–15) b) exercise: three supervised sessions in first week, one supervised and two non-supervised sessions in weeks 2–15, aerobic training (high-intensity level (60–85% of HRR)) (three 25 min sessions per week)	c) = b) low-intensity level (20–40% of HRR) + a)	Baseline, end of intervention, 3- and 6-months post-target quit date	seven-day-point-prevalence abstinence rate, CO concentration, saliva cotinine, Mini International Neuropsychiatric Interview, TND, ASI-3, PROMIS-anxiety 6 SF, PROMIS-depression 6 SF
Vickers et al. [58] RCT	n = 60 (EX: 30, CO: 30) Female adults with depression Age: 41.4 years	10-week individual tailored program: a) Smoking cessation counseling: brief counseling and handouts (one session per week, 10 min) and nicotine patch therapy (week 4–10) b1) exercise counseling sessions based on social cognitive therapy (one 30 min session per week) b2) participants could use environments that were most convenient, enjoyable and feasible for exercise completion, participants recorded daily PA	c) health education + a)	Baseline, end of intervention, 24-week follow-up	Number of cigarettes, seven-day point prevalence abstinence rate, CO concentration, urine cotinine, FTND, HRSD, CES-D, PANAS, treadmill test (maximal oxygen consumption), 7D-PAR

PA, Physical Activity; FTND, Fagerström Test for Nicotine Dependence; SEQ-12, Smoking Self-Efficacy Questionnaire; Q-MAT, Smoking cessation motivation questionnaire; HADS, Hospital Anxiety and Depression Scale Depression; BCTs, behaviour change techniques; TCQ-12, Tobacco Craving Questionnaire; IPAQ-SF, International Physical Activity Questionnaire – Short Form; SF-12, Short Form Health Survey; PSPP, Physical Self-Perception Profile; 6MWT, 6-Minute Walk Test; PHQ-9, Patient Health Questionnaire; CBT, cognitive behavioural therapy; ASI-16, 16-item Anxiety Sensitivity Index; ASI-3, Anxiety Sensitivity Index-3; IDAS, Inventory of Depression and Anxiety Symptoms; HRR, heart rate reserve; PROMIS, Patient-Reported Outcomes Measurement Information System; SF, Short Form; TND, test for nicotine dependence; HRSD, Hamilton Rating Scale for Depression; CES-D, Center for Epidemiological Studies Depression Scale; PANAS, Positive and Negative Affect Scale; 7D-PAR, Blair 7-day Physical Activity Recall

The sample size of the studies ranged from twelve [54] to 150 participants [57] with a total number of 458 participants in the included studies. Four studies recruited both sexes [54–57], and two studies were limited to female participants [42, 58]. Three of the included studies targeted people with depressive disorders [42, 55, 58], one trial involved individuals with schizophrenia or schizoaffective disorder [54], one study involved people with elevated anxiety sensitivity (score ≥ 20 on Anxiety Sensitivity Inventory (ASI-16)) [56], and one study recruited people with high anxiety sensitivity (score ≥ 23 on Anxiety Sensitivity Index-3 (ASI-3)) [57]. The indicated number of cigarettes was at least ten cigarettes per day in three studies [42, 56, 58], at least five cigarettes in the study of Smits et al. [57], and at least 15 cigarettes in the study of Bernard et al. [54]. In the study of Bernard et al. [55], participants were included if they scored four or higher in the Fagerström Test for Nicotine Dependence (FTND). Five of the six included studies recruited sedentary, low active persons, or people who were not engaged in PA on a regular basis [42, 55–58].

All six included studies used a behavioural approach including counselling for smoking cessation while five studies [42, 54–58] combined behavioural smoking cessation treatment with nicotine replacement therapy (NRT). In four studies [42, 56–58], the participants received nicotine patches and one study [55] provided patches, gums, or varenicline. In five of the six included studies, a quit date was scheduled. Depending on the study, the quit date was scheduled at different times: within 2 weeks [55], first session of the third week [42], the sixth week [56, 57], and the fourth week [58].

Different exercise modalities were offered in the included studies. Bernard et al. [54] provided supervised walking sessions. The exercise intervention of Bernard et al. [55] consisted of supervised stationary cycle ergometers and home exercise sessions of walking, cycling, or running. The exercise programme of Patten et al. [42], an aerobic activity, was conducted at a worksite fitness center and a Young Men’s Christian Association (YMCA) setting. Smits et al. [56] offered treadmill sessions. The aerobic exercise programme of Smits et al. [57] also took place in a YMCA and had a high intensity. In the study of Vickers et al. [58], participants received exercise counselling sessions with the aim of increasing activity levels. Instead of supervised exercise, participants were able to use comfortable, enjoyable, and feasible environments to perform exercise [58]. Participants recorded their daily PA and the counsellors recorded participants´ progress [58].

In the control condition of four RCTs, the participants received a wellness [56] or health education programme [42, 55, 58]. Information on a variety of health topics including sleep hygiene, stress, or nutrition was given to the participants [55, 58] and/or was discussed [42, 55, 56] and presented through lectures, handouts, or films [42, 55]. In one study [57], participants in the control group also received an exercise programme as in the intervention group, but on a low-intensity level.

In three included studies, the smoking cessation programme and the exercise and control interventions were offered as a group programme [54–56]. The other three studies provided individual sessions [42, 57, 58]. In all included RCTs, both interventions (exercise and control) were equivalent in terms of time and duration. The duration of the interventions varied between eight [54, 55], ten [58], twelve [42], or 15 weeks [56, 57]. In two studies, the interventions consisted of one session per week [54, 58]. Table 1 gives a detailed overview on the exact duration of the intervention sessions.

In four studies [42, 56–58], participants were compensated with money for completed assessments or for the participating in the entire programme. Participants in the study of Patten et al. [42] also received a free six-month gym membership. Participants in the other included studies [54, 55] received no compensation or incentives for participating in the study.

### Risk of bias

As shown in Table 2, two RCTs [42, 55] were rated to be at low risk of bias. Two studies were rated as having some concerns [57, 58] and one study as a high risk of bias study, as one domain was defined to be at high risk [56]. The domain “missing outcome data” was classified as some concerns in the study of Vickers et al. [58] due to a high drop-out rate after the end of treatment and after 24 weeks of follow-up. As the drop-out rate was almost the same in both treatment groups and an intention-to-treat analysis was performed, classifying missing data as ‘smoking’, this domain was rated with some concerns. In contrast, in the study of Smits et al. [56], the domain “missing outcome data” was judged to be at high risk. In addition to a high drop-out rate at the end of treatment and follow-ups, a generalized linear mixed models analysis was executed, with missing data not coded as smoking. The domain “selection of the reported result” was rated with some concerns in the study of Vickers et al. [58]. There is no study protocol or registration to compare predefined analysis intentions with reported outcomes. As smoking abstinence rates were reported at all assessment points and no significant results were reported, the domain was classified with some concerns. In the study of Smits et al. [57] the domain “selection of the reported result” was also classified as some concerns. Although measurements were taken at five predefined time points, the study focused on the results of 6-month follow-up, and the results of the other measurement time points were underreported.

**Table 2:**
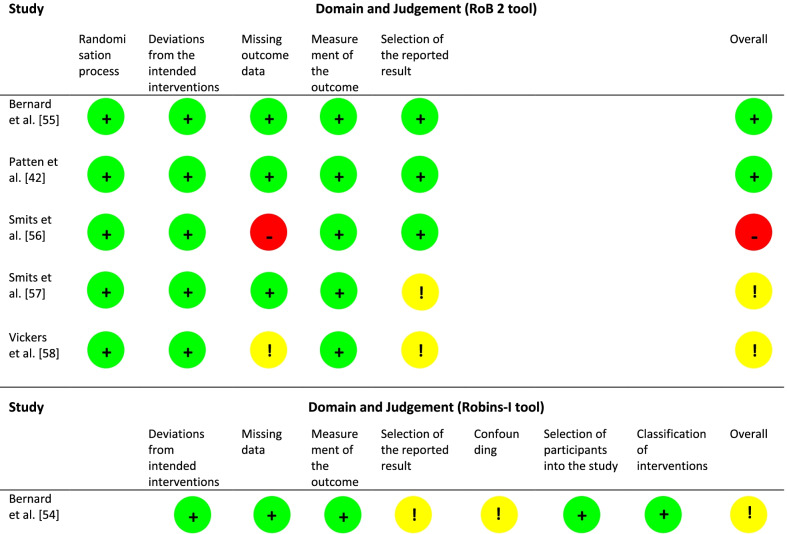
Risk of bias

The study of Bernard et al. [54] was judged to be at moderate risk of bias because two domains were classified as moderate risk (see Table 2). The domain “confounding” was rated as moderate risk. Although the adherence rate, sociodemographic variables, smoking behaviour and history, stages of change and medication change were considered and documented by Bernard et al. [54], other extraneous events or unexpected changes could have influenced the outcome of the small sample (n = 12) with schizophrenia or schizoaffective disorder. The domain “selection of the reported result” was also classified as moderate risk, because no study protocol is available to compare the reported results with predefined analyses.

### Primary outcomes

Two studies [55, 58] reported no significant differences in smoking abstinence rates between the intervention and control groups at all assessment points. In the study of Patten et al. [42], the intervention group showed significantly higher abstinence rates at the end of the intervention but not at 6-month follow-up. Smits et al. [56] divided the sample into individuals with high and low Anxiety Sensitivity Inventory (ASI) scores. The smoking abstinence rate of participants with high ASI scores was significantly higher in the exercise group than in the control group at all assessment points, but not in participants with low ASI scores [56]. In the single-group study of Bernard et al. [54], significant reduction in tobacco use and expired CO (carbon monoxide) levels after the intervention (after eight weeks) compared to baseline were shown. Smits et al. [57] observed a higher smoking abstinence rate in the intervention group (high-intensity exercise) than in the control group (low-intensity exercise) after six month follow-up, but difference between the groups was only approximately significant.

Four RCTs [42, 55, 56, 58] comparing the abstinence rates of the intervention and control groups at the end of treatment and at 6-month follow-up were included in the meta-analysis. The RCT of Smits et al. [57] was excluded because the intervention and also the control group received an exercise programme but with different intensities.

**Fig. 2:**
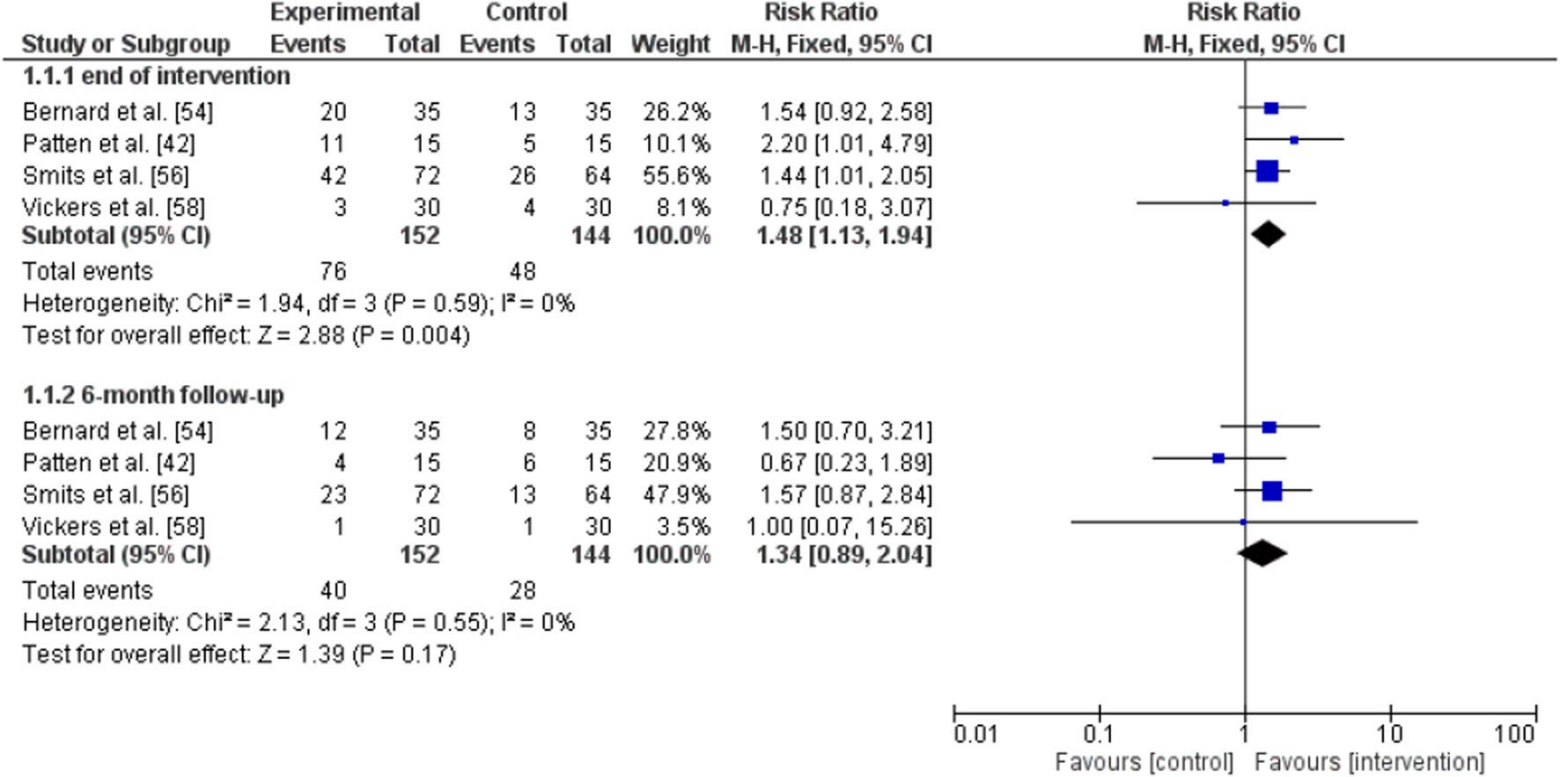
Forest plot of the meta-analysis comparing the effect of the experimental (exercise + smoking cessation) and control (health education + smoking cessation) intervention; outcome: smoking abstinence; subgroup: assessment point

The analysis for the end of the intervention showed a pooled RR of 1.48 (95% CI 1.13–1.94) indicating a significantly higher abstinence rate of the smoking cessation programme combined with an exercise intervention on smoking abstinence rate compared to the control intervention (p = 0.004). There was no evidence of heterogeneity (I^2^ = 0%). No significant overall effect (p = 0.170) of the intervention condition on smoking abstinence rates was observed for the 6-month follow-up assessment point (RR 1.34, 95% CI 0.89–2.04). There was no evidence of heterogeneity for the 6-month follow-up assessment (I^2^ = 0%) (see Fig. 2).

### Secondary outcomes

#### Treatment adherence

Considering the total intervention participation of the RCTs [42, 55–58], participants in the exercise intervention attended a total of 83.3 of 146 sessions offered (57%) during the intervention phase. The participants in the control group attended a total of 88.9 of 146 sessions offered (61%). In two studies [42, 55], treatment adherence was higher in the exercise groups than in the control groups, but with no significant difference. Vickers et al. [58] observed an opposite trend, but without significant difference. In the study of Smits et al. [56], the control group attended significantly more sessions than the exercise group. Smits et al. [56] also found a significant positive correlation between attendance rate and point prevalence abstinence as well as prolonged abstinence during the quit week. In the single-group study of Bernard et al. [54], participants attended on average over 80% of the entire programme. Smits et al. [57] found no difference in the number of training sessions completed between groups.

#### Smoking cessation motivation and smoking self-efficacy

Bernard et al. [54] reported a significant increase in motivation to quit smoking at the end of treatment compared to baseline measurements. In the same study, no significant differences in smoking self-efficacy between the two assessment points could be observed. In the study of Bernard et al. [55], no significant group differences in smoking self-efficacy were found but an increase in self-efficacy scores in both groups over time.

#### Physical activity

In the study of Bernard et al. [55], the exercise group achieved a significantly larger distance in the 6-Minute Walk Test at the end of intervention compared to the control. Measurements of objective physical activity by accelerometer on 7 consecutive days before and after the programme showed no differences between the exercise and control group and over time [55]. According to Patten et al. [42], maximal oxygen consumption was significantly higher in the intervention group than in the control group at the end of treatment. The outcomes for objective physical activity measured by accelerometer were not significantly different for both groups at the end of treatment compared to pre-treatment [42]. In the study of Vickers et al. [58], the exercise group significantly increased their self-reported physical activity at the end of treatment and at week 24 compared to the control condition. VO2 max assessed by treadmill exercise test did not differ between groups and did not improve over time in both groups [58].

#### Change in mental health symptoms

Two studies [56, 58] found a significant change in symptoms between groups. However, contradictory results were found: In the study of Vickers et al. [58], participants in the control group showed a significantly greater reduction in depression scores at week ten than participants in the exercise group. In contrast, in the study of Smits et al. [56], ASI scores and symptoms of anxiety and depression were significantly lower in the exercise group than in the control group during the quit week (week six). Smits et al. [56] also reported that lower anxiety scores were positively associated with smoking cessation success. In the study of Smits et al. [57], anxiety sensitivity, anxiety, and depression symptoms decreased significantly from baseline to quit week in both groups, but no difference was found between the intervention and control group. Patten et al. [42] also found no difference in depressive symptoms between both groups at the end of treatment. However, the symptoms of depression changed positively in both groups after treatment compared to pre-treatment [42]. Bernard et al. [55] observed a positive change in anxiety and depression symptoms in the exercise and control group after the treatments but without significant difference. In addition, no group differences were found over all measurement points [55]. In the single group study of Bernard et al. [54], depression symptoms improved after the intervention, but without significant difference. Anxiety symptoms did not change after treatment compared to pre-treatment [54].

#### Change in affective states

In the study of Vickers et al. [58], changes in affective states were measured using the PANAS (Positive and Negative Affect Schedule). Positive and negative affect states improved in both groups over time [58]. However, a significant difference in reduction of negative affect states was found only in the exercise group at week 24 compared to baseline [58]. Vickers et al. [58] observed no differences in affective states between the exercise and control group.

## Discussion

The aim of this systematic review and meta-analysis was to evaluate the effectiveness of exercise interventions as an add-on therapy to smoking cessation in people with MI. Based on the meta-analysis of four RCTs with 296 participants, the present study suggests a beneficial effect of exercise interventions as an aid to smoking cessation at the end of the intervention compared to control interventions. The effect did not reach significance at the 6-month follow-up, suggesting a temporary effect of add-on exercise interventions on abstinence rates in people with MI.

The present findings in people with MI are partly similar to previous meta-analyses on exercise interventions in people without MI. Ussher et al. [27] included 21 RCTs in their meta-analysis, with 15 of the studies targeting the general population and six targeting special groups (people with mental health issues, with physical health conditions, or with pregnancy). The meta-analyses of Ussher et al. [27] found no evidence of an effect of the exercise intervention on smoking cessation at longest follow-up (≥ 6 months) for all included studies (RR 1.08, 95% CI 0.96–1.22, I^2^ = 0%) and in the sensitivity analysis excluding the six studies with special groups (RR 1.06, 95% CI 0.92–1.22, I^2^ = 0%). These results are consistent with our findings from the meta-analysis that examined the effect at the 6-month follow-up. In the meta-analysis of Klinsophon et al. [26], the effect of exercise and type of exercise on smoking cessation at the end of treatment and at the end of follow-up in individuals without MI were examined. Klinsophon et al. [26] reported no significant effect of exercise (RR 1.13, 95% CI 0.94–1.35, 13 RCTs), and aerobic exercise (RR 1.13, 95% CI 0.89–1.44, 9 RCTs) on the point prevalence abstinence rate at the end of treatment in persons without MI. In contrast to these findings, our meta-analysis found an overall effect at the end of treatment in people with MI. However, a subgroup analysis of Klinsophon et al. [26] is consistent with our findings. Klinsophon et al. [26] found a positive effect of yoga on point prevalence abstinence rates at the end of treatment in people without MI (RR 3.11, 95% CI 1.00–9.69, 1 RCT). One possible explanation is that the components of yoga, breathing exercise and mediation, cause a decrease in sympathetic activity and an increase in parasympathetic activity, and lead to relaxation and stress reduction [26]. Both, our meta-analysis and the meta-analysis by Klinsophon et al. [26], showed no long-term effects of exercise on smoking abstinence rates at the end of follow-up.

The following differences from the previous findings for people without MI can be identified: The similarity of control interventions appears to be specific to people with MI. In the four included RCTs [42, 55, 56, 58] in the present review, the content of the control programmes was very similar, as all participants in the control group received a health education programme. Studies in people without MI [60] show considerable heterogeneity in control interventions. In addition, people with MI show relatively low adherence to exercise and smoking cessation programmes compared to individuals without MI [31, 61, 62]. The results of the present review support this statement, as the overall adherence of all RCTs was 53% in the intervention group and 61% in the control group among people with MI. Several factors, such as higher nicotine dependence, greater number of cigarettes per day, longer smoking history, low self-confidence and self-efficacy, and lower motivation to quit, are associated with lower participation and higher drop-out rates in exercise and smoking cessation programmes [61]. These factors may apply to a greater number of people with MI than those without MI [15, 18]. Regardless of group assignment, Smits et al. [56] demonstrated a significant association between higher participation and increased smoking abstinence [56]. Therefore, efforts to increase adherence to exercise are recommended, such as informing patients about the importance of adherence, motivational support during the programme, financial incentives, cost-effective programmes, and programmes that fit into existing care structures of people with MI [6, 31, 63].

Although the effect of exercise interventions on abstinence rates seems to be rather small and temporary, potential benefits for secondary outcomes should also be considered. Similar to people without MI [27], we observed an increased physical activity level at the end of treatment among people with MI [42, 55, 58] in the present review. We could also see, that participants improved their symptom severity, with this being observed in the control group in one study [58] and in the exercise group in two studies [56, 57]. As there are associations between psychotic disorders and adverse health behaviours and risk factors for cardiovascular disease such as insufficient physical activity, poorer diet, and obesity [4, 5, 64–66], exercise interventions could not only help to reduce smoking, but also to improve cardiovascular health and quality of life [67].

Since the challenge to quit smoking might be higher for individuals with MI [14, 15, 17, 18], it is important, that people with MI are offered a wide range of different and appropriate approaches and strategies for smoking cessation. Therefore, it is necessary that various responsible organisations such as research institutes, medical centers, and other healthcare organisations contact people with MI, provide information about these approaches, and offer additional smoking cessation programmes [31]. Since psychologists, physicians and medical staff work with individuals with MI in medical centers and healthcare organisations, they have the opportunity of interacting with them and can inform them about the benefits of quitting or reducing smoking and about offers. Research institutes can communicate smoking cessation studies for people with MI through medical centers.

Smoking cessation programmes that include pharmacological and behavioural interventions for people with MI are well established. A current meta-analysis of Peckham et al. [6] found a significant effect of adding bupropion to smoking cessation programmes on quit rates in medium (Mdn = 3.5 months) and long term (Mdn = 11.75 months), but not in the short term (Mdn = 4 weeks) in people with MI (short term RR 6.42, 95% CI 0.82–50.07, I^2^ = 0%; medium term RR 2.93, 95% CI 1.61–5.34, I^2^ = 0%; long term RR 3.04, 95% CI 1.10–8.42, I^2^ = 0%). The addition of varenicline significantly improved quit rates in the medium term (Mdn = 6 months) in people with MI (RR 4.13, 95% CI 1.36–12.53, I^2^ = 0%) [6]. No evidence of the benefit of behaviour change techniques in smoking cessation counselling in people with MI in medium (Mdn = 6 months) and long term (Mdn = 12 months) could be seen (medium term RR 1.32, 95% CI 0.85–2.06, I^2^ = 70%; long term RR 1.33, 95% CI 0.85–2.08, I^2^ = 0%) [6]. Compared with behavioural and pharmacological interventions for smoking cessation in persons with MI, exercise interventions in smoking cessation in people with MI is a relatively new area of research that has developed over the past 12 years. Given the lack of evidence in this population, the present study may foster research in this field.

In addition, it should be noted that smoking cessation and exercise programmes are often similar for people without and with MI. More attention should be paid to the individual needs of people with MI, in terms of programme content and outcome [15, 19]. With regard to the content of exercise and smoking cessation programmes, individual preferences regarding modality, duration, and intensity should be considered [66]. An example of this is the study of Smits et al. [57], which showed a higher abstinence rate in the high-intensity exercise group in people with MI compared to the low-intensity exercise control group. In addition to the main outcome of a smoking cessation programme of reducing or quitting smoking, other individual outcomes of people with MI should also be considered. Experiences of success, such as improvements in well-being, affective states, health behaviours, and physical activity, as well as reductions in symptom severity, can have a positive impact on self-confidence and the ability to quit smoking [19].

The informative value of the present meta-analysis and the outcomes in the systematic review are limited by the following aspects. The most important limitation is the small number of included studies and the small sample sizes of the original studies. Therefore, no subgroup or moderator analyses were conducted. In particular, analyses regarding exercise modality, duration, incentives for participants, or programme setting (group or individual), would have given a more detailed insight into the ideal characteristics of exercise interventions. In addition, if the number of studies had been large enough, it would have been interesting to divide the studies by mental disorders. People with certain disorders, such as bipolar disorder and schizophrenia, have high smoking rates, which may lead to different abstinence rates [68, 69]. Due to a limited number of studies in the literature investigating add-on exercise interventions to smoking cessation in people with MI, the authors of this review also included two studies that examined individuals with elevated anxiety sensitivity [56, 57]. Since elevated anxiety sensitivity may be related to emotional disorders and thus may indicate a disorder [47], and since smoking is more prevalent in individuals with elevated anxiety sensitivity compared with the general population [44–46], these studies were included in our review. It should also be noted that the studies included in the meta-analysis are only comparable to a limited extend. Although they were similar in terms of smoking cessation programmes, control interventions and physical activity status of participants, they differed in terms of exercise programmes and intervention duration (8–15 weeks).

Due to the small number of eligible studies, one study was included in the meta-analysis, that was judged to be at high risk of bias. This particular study was accepted, because only one domain “missing outcome data” of the overall study judgement was classified as high risk due to the high drop-out rate and the data analysis. The different domains of the risk of bias assessment could be classified well for all studies, as all studies provided sufficient information on relevant aspects like randomisation process, intervention groups, outcome analyses and results.

Limitations in the search and selection process were reduced as far as possible by using appropriate and established approaches such as software programmes and PRISMA-checklist [43]. Despite detailed searches of relevant databases, unpublished papers, trial registries, ongoing studies, and reference lists of previous reviews [19, 26, 27, 70–72], we may still have missed potentially important studies. All titles and abstracts were screened individually, and only studies in English and German were included.

## Conclusion

To the best of our knowledge, this is the first systematic review and meta-analysis that investigated the efficacy of exercise interventions as an aid to smoking cessation in people with MI. According to the current state of research, exercise in combination with smoking cessation programmes can help not only to reduce smoking but also to improve mental health symptoms, physical activity, and cardiovascular health. Therefore, health care providers should consider implementing exercise interventions in addition to standard behavioural smoking cessation programmes to improve the physical and mental health of people with MI. However, the results should be viewed with caution due to the limited number of included studies with different exercise interventions and durations. As there is still little research in this area, further trials with larger samples investigating smoking cessation programmes with exercise interventions in people with MI are needed.

## Data Availability

The data supporting our findings can be found in the published manuscripts cited.

## Abbreviations

MI: Mental illness
RCT: Randomised controlled trial
RR: Risk ratio
CI: Confidence interval
PROSPERO: Prospective Register of Systematic Reviews
PA: Physical activity
PRISMA: Preferred Reporting Items for Systematic Reviews and Meta-Analyses
BMI: Body mass index
DSR: Distiller Systematic Review
RoB 2: Version 2 of the Cochrane risk-of-bias tool for randomised trials
ROBINS-I: Risk of Bias In Non-randomised Studies – of Interventions
NRSI: Non-randomised studies of interventions
ITT: Intention-to-treat
FTND: Fagerström Test for Nicotine Dependence
NRT: Nicotine replacement therapy
YMCA: Young Men´s Christian Association
ASI: Anxiety Sensitivity Inventory
CO: Carbon monoxide
SD: Standard deviation
Mdn: Median
